# DDX3 promotes tumor invasion in colorectal cancer via the CK1ε/Dvl2 axis

**DOI:** 10.1038/srep21483

**Published:** 2016-02-19

**Authors:** Tsung-Ying He, De-Wei Wu, Po-Lin Lin, Lee Wang, Chi-Chou Huang, Ming-Chih Chou, Huei Lee

**Affiliations:** 1 Institute of Medicine, Chung Shan Medical University, Taichung, Taiwan; 2 Graduate Institute of Cancer Biology and Drug Discovery, Taipei Medical University, Taipei, Taiwan; 3 School of Public Health, Chung Shan Medical University, Taichung, Taiwan; 4 School of Medicine, Chung Shan Medical University and Hospital, Taichung, Taiwan; 5 Department of Surgery, Chung Shan Medical University and Hospital, Taichung, Taiwan

## Abstract

DDX3, a subunit of CK1ε, phosphorylates Dvl2 to promote β-catenin activation. Overexpression of the Dvl2 protein results in potent activation of β-catenin/TCF signaling in colorectal cancer. Therefore, we hypothesized that DDX3 might promote tumor invasion via the CK1ε/Dvl2 axis due to β-catenin/TCF activation. Western blotting showed that β-catenin expression was decreased by DDX3 knockdown and increased by DDX3 overexpression in colorectal cancer cells. The TCF promoter activity and invasion capability were concomitantly increased and decreased by DDX3 manipulation in these cells. The invasion capability in colon cancer cells and xenograft lung tumor nodules induced by a DDX3-overexpressing T84 stable clone in tail-vein injection model were nearly suppressed by inhibitors of CK1ε (PF4800567) and β-catenin/TCF signaling (XAV939). Among colorectal cancer patients, DDX3 expression was positively correlated with the expression of pDvl2 and nuclear β-catenin in tumor tissues. The expression of pDvl2 occurred more frequently in high-nuclear than in low-nuclear β-catenin tumors. A prognostic significance of DDX3, pDvl2, and nuclear β-catenin on overall survival and relapse free survival was observed in this study population. We therefore suggest CK1ε or β-catenin/TCF signaling as potential targets for improving tumor regression and outcomes in colorectal cancer, particularly tumors with high-DDX3/high-nuclear β-catenin or high-DDX3/high-pDvl2/high-nuclear β-catenin expression.

Wnt/β-catenin signaling plays a critical role in embryogenesis as well as in tumorigenesis^1^. In the absence of Wnt ligands, Ser/Thr residues in the N-terminus of β-catenin undergo constitutive phosphorylation by a cytoplasmic destruction complex consisting of adenomatous polyposis coli (APC), axin, casein kinase 1α (CK1α), and glycogen synthase kinase 3β (GSK3β), which in turn facilitates ubiquitination of β-catenin by β-TrCP E3 ligase^2^. The phosphorylation of β-catenin at serine (Ser)33, Ser37, and threonine (Thr)41 by GSK3β plays a critical role in promoting β-catenin degradation^3^. The phosphorylation of GSK3β at Ser9 by the RAS/mitogen-activated protein kinase kinase (MEK)/extracellular signal-regulated kinase (ERK) and the phosphatidylinositide 3-kinase (PI3K)/AKT signaling pathways in turn plays a crucial role in suppressing GSK3β activity^4^,^5^. A protein phosphatase 2A (PP2A) also promotes β-catenin degradation and thereby inhibits Wnt/β-catenin signaling^6^, while casein kinase 1ε (CK1ε) decreases the association of PP2A with the β-catenin degradation complex^7^. An increase in β-catenin protein stability determines the levels of cytoplasmic β-catenin accumulation and nuclear β-catenin binding with the T-cell factor/lymphoid enhancer factor (TCF/LEF) or other transcription factors, thereby upregulating several downstream genes, such as cyclin D1 and c-Myc, to promote tumor progression^8^,^9^,^10^.

Dysregulation of Wnt/β-catenin signaling is therefore an initiating event underlying colon adenoma formation following the loss of APC^1^,^11^,^12^. However, the loss of APC alone is not sufficient to promote aberrant Wnt/β-catenin signaling^13^,^14^,^15^,^16^. Accumulating evidence now indicates that oncogenic KRAS or tumor microenvironmental factors might synergistically promote the Wnt/β-catenin activation mediated by APC loss^16^,^17^,^18^. Therefore, we suggest that some mechanism(s) other than APC mutation could be involved in activation of the β-catenin/TCF signaling during colorectal tumorigenesis.

DDX3, a DEAD-box RNA helicase, has been identified as a regulator of the β-catenin/TCF signaling that acts as a regulatory subunit of CK1ε to promote phosphorylation of disheveled segment polarity protein 2 (Dvl2). A requirement for DDX3 has been suggested for β-catenin activation during the development of mammalian cells^19^. A recent report indicated that inhibition of DDX3 by RK-33, an inhibitor of DDX3, caused G1 cell cycle arrest, induced apoptosis, and promoted tumor regression in lung cancer via disruption of the DDX3-β-catenin axis; however, the underlying mechanism of β-catenin activation by DDX3 was not mentioned^20^. Interestingly, DDX3 modulates cell adhesion and motility in HEK293 embryonic kidney cells, as well as cell invasion in HeLa and N2A cells, via the Rac1-mediated β-catenin regulatory axis^21^. DDX3 knockdown by its shRNA reduced cell proliferation and caused G1-arrest in HCT116 and HT29 colon cancer cells^22^, whereas high DDX3 expression was positively correlated with nuclear β-catenin expression in tumors from colorectal cancer patients. Our preliminary immunohistochemistry data showed that DDX3 expression was positively correlated with phosphorylated Dvl2 (pDvl2) and with high-nuclear β-catenin expression. A prognostic significance was observed for DDX3, pDvl2, and nuclear β-catenin expression on overall survival (OS) and relapse free survival (RFS) in a small subset of colorectal cancer patients. We therefore hypothesized that DDX3 could promote tumor malignancy by increasing the stability of the β-catenin protein and by promoting its translocation to the nucleus via the CK1ε/Dvl2 axis.

## Results

### DDX3 promotes cell invasion via activation of β-catenin/TCF signaling

**Figure 1:**
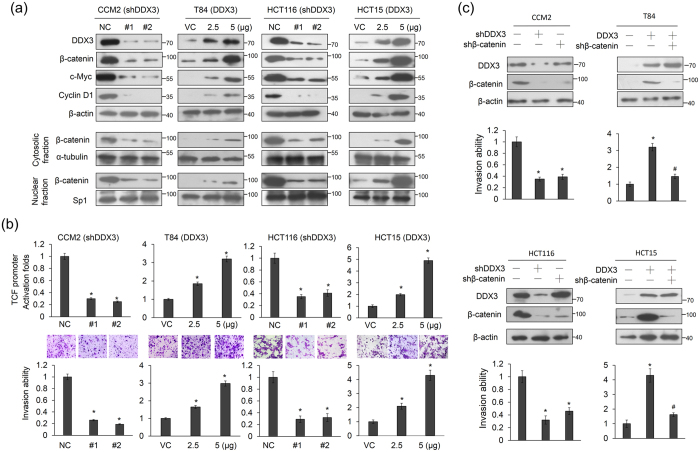
DDX3 promotes the invasion capability via β-catenin/TCF activation in colon cancer cells. (**a**) Two kinds of shDDX3 were transfected into high-DDX3-expressing CCM2 and HCT116 cell lines. Two doses of DDX3-overexpression vector were transfected into low-DDX3-expressing T84 and HCT15 cells. The total amounts of transfected shDDX3 and its expression vector were kept constant by adding the control vector. After 48 h, these cells were separated into cytosolic and nuclear fractions to evaluate β-catenin protein expression by SDS-PAGE and western blotting. The total lysates were also harvested and evaluated for levels of DDX3, β-catenin, cyclin D1, c-Myc, and β-actin protein by western blotting. NC: non-specific shRNA control. VC: empty vector control. (**b**) TCF promoter activity in DDX3 knockdown in CCM2 and HCT116 cells and DDX3-overexpressing T84 and HCT15 cells was evaluated by luciferase reporter activity assay. The invasion capability was evaluated in CCM2 and HCT116 cells with or without DDX3 shRNA transfection and in T84 and HCT15 cells with or without DDX3-overexpression vector transfection. The invasion capability in CCM2 and T84 cells with different transfections was compared with their NC and VC. (**c**) CCM2 and HCT116 cells were transfected with shDDX3 or shβ-catenin plasmids for 24 h. T84 and HCT15 cells were transfected with the DDX3 expression vector and/or co-transfected with shβ-catenin for 24 h. The expression of DDX3 and β-catenin in CCM2, HCT116, T84, and HCT15 cells subjected to moloecular manipulation were evaluated by western blotting and their invasion capability was determined by Boyden chamber assays. The invasion ability and the TCF promoter activity of these cells with different treatments were shown as the fold changes compared with their NC and VC. All experiments were performed three independent times. The mean values and the standard deviations are indicated as columns with error bars. The P value was statistically determined by the Student’s t-test. *P < 0.05 compared with vector (VC) or non-specific shRNA controls (NC). #P < 0.05 compared with DDX3-overexpressing T84 and HCT15 cells. The samples were derived from the same experiment and gels/blots were processed in parallel. Full-length blots are presented in Supplementary Figure S1.

We examined whether DDX3 could promote β-catenin activation and cell invasiveness using high-DDX3-expressing CCM2 and HCT116 cells and low-DDX3-expressing T84 and HCT15 cells to knock down and overexpress DDX3 using two shRNAs and its expression vector. As expected, DDX3 expression was significantly decreased by both shRNA transfections in CCM2 and HCT116 cells. Interestingly, expression of β-catenin and its downstream genes, cyclin D1 and c-Myc, were significantly decreased by DDX3 knockdown in CCM2 and HCT116 cells (Fig. 1a). Moreover, nuclear β-catenin expression was decreased in DDX3-knockdown CCM2 and HCT116 cells (Fig. 1a). Conversely, the opposite effects were observed on the expression of β-catenin, cyclin D1, and c-Myc in the DDX3-overexpressing T84 and HCT15 cells (Fig. 1a). A luciferase reporter assay indicated that the TCF promoter activity was markedly decreased and increased by DDX3 manipulation in CCM2, HCT116, T84 and HCT15 cells (Fig. 1b upper panel). Concomitantly, the invasion capability was decreased and increased by DDX3 manipulation in these four cell types (Fig. 1b lower panel).

We further examined whether β-catenin could be responsible for DDX3-mediated cell invasion by transfecting CCM2, HCT116, T84, and HCT15 cells with shDDX3 and its expression vector and/or with shβ-catenin. The expression levels of DDX3 and β-catenin in CCM2 and HCT116 cells with DDX3 manipulation and/or β-catenin silencing were confirmed by western blotting (Fig. 1c). The invasion capability in CCM2 and HCT116 cells was markedly decreased by DDX3 or β-catenin silencing (Fig. 1c left). Conversely, the invasion capability was increased by DDX3 overexpression in T84 and HCT15 cells, but the increase in the invasion capability by DDX3 overexpression was nearly completely reversed by β-catenin silencing (Fig. 1c right lower panel). These results suggest that the β-catenin/TCF signaling may be responsible for DDX3-mediated cell invasion.

### DDX3 activates β-catenin/TCF signaling by increasing β-catenin protein stability via the CK1ε/Dvl2 axis

**Figure 2:**
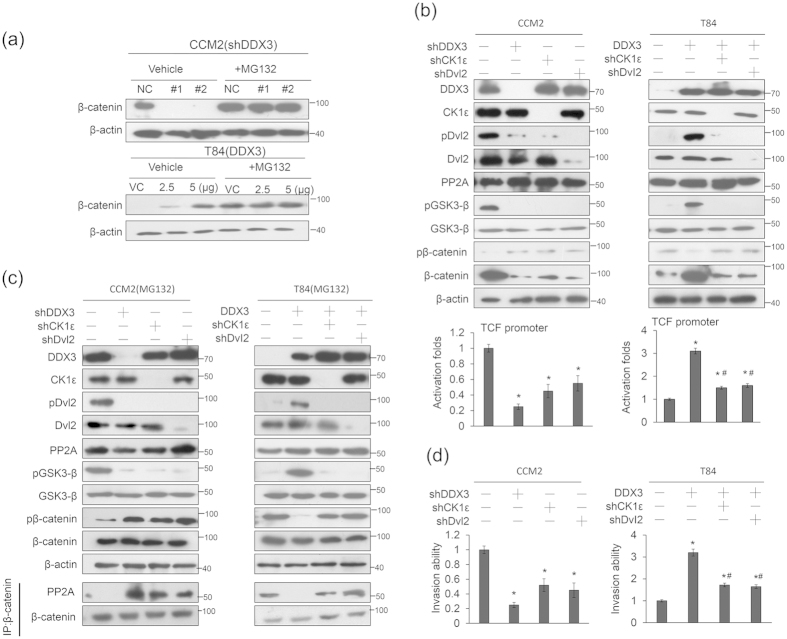
β-catenin/TCF activation by DDX3 is mediated through inhibition of β-catenin degradation by PP2A. (**a**) CCM2 and T84 cells were transfected with two kinds of shDDX3 and two doses of DDX3-overexpression vector. These cells were incubated with 5 μmol/L MG132 for an additional 5 h, and then the cell lysates were analyzed by immunoblotting with anti-β-catenin antibody. (**b**) The CCM2 cells were transfected with the indicated combination of TCF promoter plasmid, shDDX3, shCK1ε, and shDvl2 for 48 h. The T84 cells were transfected with the indicated combination of the TCF promoter plasmid, the DDX3-overexpression plasmid, shCK1ε, and shDvl2 for 48 h. The expression of DDX3, CK1ε, Dvl2, pDvl2, β-catenin, pβ-catenin (Ser33, Ser37, and Thr41), GSK-3β, pGSK-3β (Ser9), PP2A (A subunit), and β-actin were determined by western blotting using their specific antibodies. The TCF promoter activity was evaluated by a luciferase reporter activity assay. (**c**) The CCM2 cells were transfected with shDDX3, shCK1ε, or shDvl2 for 48 h. The T84 cells were transfected with the indicated combination of DDX3-overexpression plasmid, shCK1ε, and shDvl2 for 48 h. These cells were then treated with MG132 for 5 h. The cell lysates were immunoprecipitated with anti-β-catenin–conjugated beads to analyze by immunoblotting with anti-PP2A and anti-β-catenin antibody. (**d**) The CCM2 cells were transfected with shDDX3, shCK1ε, or shDvl2 for 24 h. The T84 cells were transfected with the indicated combination of DDX3-overexpression plasmid, shCK1ε, and shDvl2 for 24 h. The invasion capability of the indicated cells was determined by Boyden chamber assays. The invasion ability and the TCF promoter activity of these cells with different treatments are shown as fold changes compared with their NC and VC. All experiments were performed three independent times. The mean values and the standard deviations are indicated as columns with error bars. The P value was statistically determined by the Student’s t-test. *P < 0.05 compared with vector (VC) or non-specific shRNA controls (NC). #P < 0.05 compared with DDX3-overexpressing T84 cells. The samples were derived from the same experiment and gels/blots were processed in parallel. Full-length blots are presented in Supplementary information Figure S1 and S2.

We examined the possibility that DDX3 could activate β-catenin/TCF signaling via the CK1ε/Dvl2 axis through increases in β-catenin protein stability. The CCM2 and T84 cells were transfected with shDDX3 and its expression vector, respectively, with or without treatment with a proteasome inhibitor MG132. Western blotting showed that the disappearance of β-catenin expression in DDX3-knockdown CCM2 and T84 NC cells was reversed by MG132 treatment (Fig. 2a). The role of the CK1ε/Dvl2 axis in activation of β-catenin/TCF signaling was further examined by cotransfecting DDX3-knockdown CCM2 and DDX3-overexpressing T84 cells with shCK1ε or shDvl2. Western blotting indicated that the expressions of CK1ε, Dvl2, and PP2A were essentially unchanged by DDX3 knockdown, but the expressions of pDvl2 and β-catenin were markedly decreased in the DDX3-knockdown CCM2 cells (Fig. 2b left upper panel). In addition, the expressions of pDvl2 and β-catenin were markedly decreased by CK1ε or Dvl2 silencing in the CCM2 cells. The TCF promoter activity was concomitantly reduced by DDX3 silencing, but the decrease in the TCF promoter activity by shCK1ε or shDvl2 transfection did not exceed that achieved with DDX3 silencing in CCM2 cells (Fig. 2b left lower panel). Conversely, the expression of pDvl2 and β-catenin was significantly increased by DDX3 overexpression in T84 cells, but the increases in both pDvl2 and β-catenin were suppressed by CK1ε or Dvl2 silencing (Fig. 2b right upper panel). The TCF promoter activity was significantly increased by DDX3 overexpression, but the increase was suppressed by CK1ε or Dvl2 silencing (Fig. 2b right lower panel). The decrease in β-catenin due to DDX3 knockdown was overcome by MG132 treatment, but pDvl2 expression was unchanged in CCM2 cells after MG132 treatment (Fig. 2c upper left panel). Similar findings were observed in DDX3-overexpressing T84 cells following MG132 treatment (Fig. 2c upper right panel).

Immunoprecipitation (IP) analysis further showed that the interaction between PP2A and β-catenin was observed in CCM2 cells with shDDX3, shCK1ε, or shDvl2 transfections in the presence of MG132 (Fig. 2c bottom left panel). Conversely, the interaction between PP2A and β-catenin disappeared in DDX3-overexpressing T84 cells, but this interaction was almost completely restored in DDX3-overexpressing T84 cells by shCK1ε or shDvl2 transfection (Fig. 2c bottom right panel). In addition, DDX3 overexpression inhibited PP2A interaction with β-catenin in a dose-dependent manner in colon cancer cells (Supplementary Figure S3). We therefore suggest that activation of the CK1ε/Dvl2 axis by DDX3 plays a crucial role in β-catenin protein stability and its signaling activation.

Mechanistically, an increase in the interaction of PP2A with β-catenin, due to silencing of DDX3, CK1ε, or Dvl2, would promote β-catenin phosphorylation through the activation of GSK3β due to GSK3β dephosphorylation at Ser9 (Fig. 2b,c). These results support a previous study indicating that a decrease in the interaction between PP2A and β-catenin due to DDX3 overexpression may confer β-catenin protein stability^7^. Boyden chamber assays indicated that the invasion capability markedly decreased in the CCM2 cells and markedly increased in the T84 cells in response to DDX3 manipulation. In addition, the reduction of invasion capability by CK1ε or Dvl2 silencing was revealed in CCM2 and DDX3-overexpressing T84 cells (Fig. 2d). However, the invasion capability modulated by CK1ε or Dvl2 silencing did not exceed that seen in the DDX3-knockdown CCM2 cells (Fig. 2d). These results suggest that the activation of β-catenin/TCF signaling mediated by DDX3 may occur at least in part through the CK1ε/Dvl2 axis.

### The cell invasion in DDX3-overexpressing T84 cells and in lung tumor nodules in nude mice induced by a DDX3-overexpressing T84 stable clone can be suppressed by an inhibitor of CK1ε or β-catenin/TCF signaling

**Figure 3:**
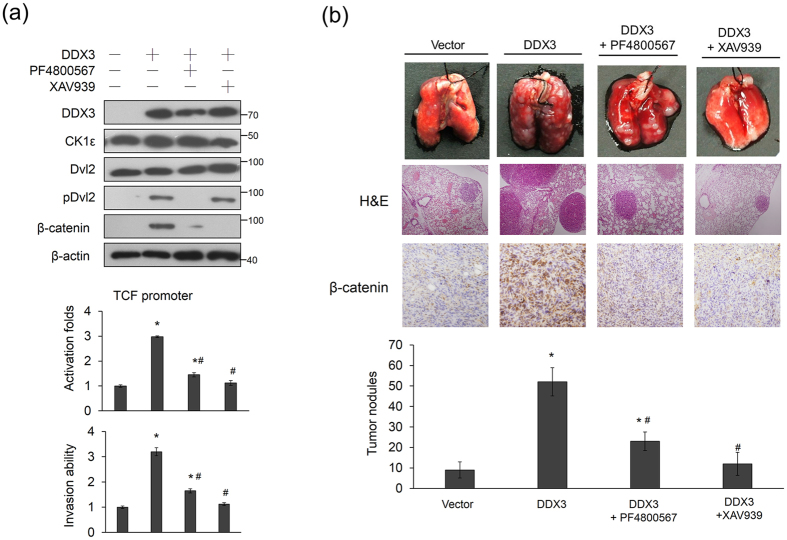
DDX3-mediated β-catenin/TCF activation is responsible for invasiveness in colon cancer cells and for lung tumor nodules formation in nude mice. (**a**) T84 cells were transfected with the indicated combination of TCF promoter plasmid and DDX3-overexpression plasmid. After 24 h, these cells were treated with a CKlε inhibitor (PF4800567) or a β-catenin inhibitor (XAV939) for an additional 5 h, and then the invasion capability for indicated cells was determined by Boyden chamber assays. The TCF promoter activity was evaluated by a luciferase reporter assay. The invasion ability and the TCF promoter activity of these cells with different treatments were shown as the fold changes compared with their NC and VC. All experiments were performed three independent times. The mean values and the standard deviations are indicated as columns with error bars. The P value was statistically determined by the Student’s t-test. *P < 0.05 compared with vector (VC) or non-specific shRNA controls (NC). #P < 0.05 compared with DDX3-overexpressing T84 cells. (**b**) Examples of lungs of mice with visual lung tumor nodules at 6 weeks after tail vein inoculation of indicated cells. Representative hematoxylin and eosin staining demonstrates the lung tumor nodules from each group of mice. The number of lung tumor nodules in each group of mice is shown. β-catenin expressions in lung tumor nodules were evaluated by immunohistochemistry using a specific antibody. Data are presented as means ± SD. The P value was statistically determined by the Student’s t-test. *P < 0.05 compared with vector control. #P < 0.05 compared with DDX3-overexpressing T84 cells. The samples were derived from the same experiment and gels/blots were processed in parallel. Full-length blots are presented in Supplementary information Figure S2.

We next examined whether an inhibitor of CK1ε or of β-catenin/TCF signaling could inhibit the cell invasion capability induced by DDX3 overexpression in T84 cells. Western blotting showed that pDvl2 expression was markedly decreased by treatment of the DDX3-overexpressing T84 cells with the CK1ε inhibitor PF4800567. β-catenin expression was markedly decreased by treatment with PF4800567 or with a β-catenin/TCF signaling inhibitor (XAV939) (Fig. 3a upper panel). The TCF promoter activity and invasion capability were reduced by both inhibitors in DDX3-overexpressing T84 cells, but the inhibitory effect on both activities was slightly greater with XAV939 than with PF4800567 (Fig. 3a lower panel). The 3-(4,5-cimethylthiazol-2-yl)-2,5-diphenyl tetrazolium bromide (MTT) assay showed that cell viability was essentially unchanged by DDX3 manipulation and drug treatments at 16 h (Supplementary Figure 3). The tail-vein injection animal model showed that PF4800567 and XAV939 treatment significantly reduced the number of lung tumor nodules in nude mice injected with the DDX3-overexpressing T84 stable clone (Fig. 3b). Consistent with the cell model, the inhibitory effects on lung tumor nodules formations were greater with XAV939 than with PF4800567 (Fig. 3b). These results obtained from the animal model support the mechanistic action of the cell model and suggest that DDX3-mediated β-catenin/TCF activation occurs partially through the CK1ε/Dvl2 axis.

### DDX3 is positively correlated with pDvl2 and nuclear β-catenin expression and is associated with OS and RFS in colorectal cancer patients

**Figure 4:**
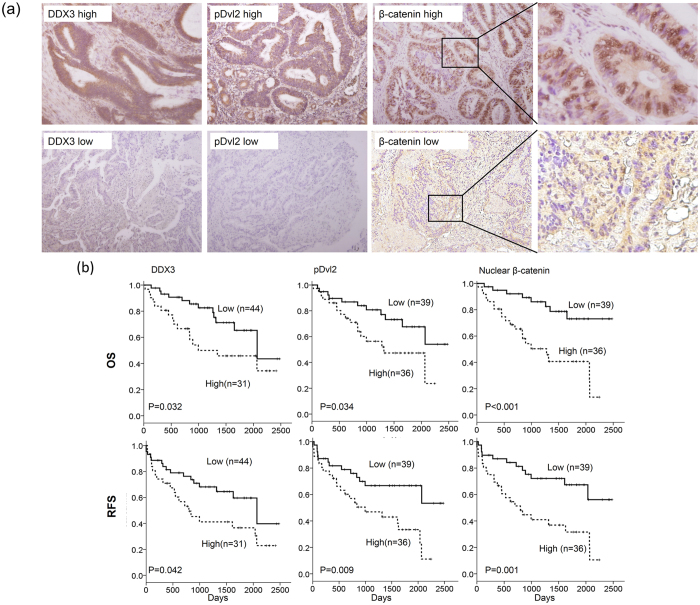
The representative immunostaining results for DDX3, pDvl2, β-catenin expression in colorectal tumors and the prognostic value of these expressions on OS and RFS was assessed by Kaplan-Meier analysis. (**a**) The representative immunostaining results of DDX3, pDvl2, and nuclear β-catenin in colorectal tumors; (**b**) The survival curves for overall survival (OS) and relapse free survival (RFS) in colorectal patients with high-DDX3, high-pDvl2, and high-nuclear β-catenin tumors were compared with those with low-DDX3, low-pDvl2, and low-nuclear β-catenin tumors.

We examined the possibility that DDX3 could be associated with phosphorylation of Dvl2 and nuclear β-catenin expression in colorectal cancer. In total, 75 tumors surgically resected from colorectal cancer patients were enrolled to evaluate DDX3, pDvl2, and β-catenin expression by immunohistochemistry. The representative immunostaining results of DDX3, pDvl2, and β-catenin are shown in Fig. 4a. High pDvl2 and high nuclear β-catenin expressions were more commonly observed in high-DDX3 tumors than in low-DDX3 tumors (71% vs. 32%, P = 0.001; 68% vs. 34%, P = 0.004; Table 1). High pDvl2 expression was almost always detected in high-nuclear β-catenin tumors (92% vs. 8%); conversely, low pDvl2 expression was frequently observed in low-nuclear β-catenin tumors (92% vs. 8%, P < 0.001; Table 1).

We next examined whether DDX3, pDvl2, and nuclear β-catenin expressions could be associated with clinical outcomes in colorectal cancer patients. Kaplan-Meier analysis showed that patients with high-DDX3, high-pDvl2, and high-nuclear β-catenin tumors had shorter OS and RFS periods when compared to patients with low-DDX3, low-pDvl2, and low-nuclear β-catenin tumors (Fig. 4b). Cox regression analysis further indicated a prognostic significance of these three molecules on OS and RFS in this study population (Table 2). The hazard ratios of high-DDX3, high-pDvl2, and high-β-catenin tumors were 2.57, 3.07, and 3.97 for OS and 2.26, 2.79, and 2.96 for RFS, respectively, when low-DDX3, low-pDvl2, and low-nuclear β-catenin tumors was used as the reference (Table 2). Moreover, high-DDX3/high-pDvl2, high-DDX3/high-nuclear β-catenin, and high-DDX3/high-pDvl2/high-nuclear β-catenin tumors had higher HR value for OS and RFS when compared with high-DDX3, high-pDvl2, and high-nuclear β-catenin tumors (4.55, 5.61, and 4.78 for OS; 2.71 2.67, and 2.41 for RFS, respectively; Table 2). These results suggest that DDX3-induced pDvl2 and nuclear β-catenin expressions may promote tumor malignancy and consequently result in poor outcomes in colorectal cancer patients.

## Discussion

The evidence provided here to demonstrate that β-catenin/TCF activation due to increased β-catenin protein stability may be responsible for DDX3-mediated cell invasion and xenograft lung tumor nodules formation via the CK1ε/Dvl2 axis. Consistent with a previous study, the interaction between PP2A and β-catenin can be blocked by Dvl2 phosphorylation, which destabilizes the β-catenin degradation complex^7^ (Fig. 2c). DDX3 is known to promote HeLa cell invasion via Rac1-mediated β-catenin activation^21^. In the present study, DDX3-mediated β-catenin activation occurred through the CK1ε/Dvl2 axis and, in turn, promoted tumor invasion and poor prognosis in colorectal cancer patients. Therefore, Dvl2 phosphorylation by CK1ε and/or DDX3 would appear to play a central role in cytoplasmic β-catenin accumulation and its nuclear translocation, and consequently in the promotion of tumor invasion in colorectal cancer patients.

Dvl2 promotes self-renewal and tumorigenesis in human gliomas via activation of Wnt/β-catenin signaling^23^. Overexpression of Dvl2 proteins can act as a potent activator of the β-catenin/TCF signaling^24^. Deletion of Dvl2 reduced the number of intestinal tumors formed in the APC^Min^ mouse model^24^. Our immunohistochemical data indicated that high-nuclear β-catenin expression occurred more frequently in high-pDvl2 tumors than in low-pDvl2 tumors (Table 1). This result strongly supports the finding of a previous study^24^, where overexpression of pDvl2 was positively correlated with nuclear β-catenin expression in colorectal cancer. To the best of our knowledge, the present study is the first to show that pDvl2 expression independently predicts poor outcomes in colorectal cancer patients. Moreover, the prognosis for OS and RFS was worse for the combination of pDvl2 with DDX3, or with DDX3/high-nuclear β-catenin than for pDvl2, DDX3, or high-nuclear β-catenin alone (Table 2). Therefore, the evidence from the cell, animal, and human tissue studies indicated that phosphorylation of Dvl2 by DDX3 and/or CK1ε plays an important role in colorectal tumorigenesis.

The decreases in the TCF promoter activity and invasion capability were lower following DDX3 knockdown than following CK1ε and Dvl2 silencing in colon cancer cells (Fig. 2b,d). These results suggest that DDX3-mediated β-catenin/TCF activation does not occur exclusively through the CK1ε/Dvl2 axis. As mentioned above, phosphorylation of β-catenin at Ser33, Ser37, and Thr41 by GSK3β plays a central role in β-catenin degradation^3^. GSK3β activity is suppressed by phosphorylation of GSK3β at Ser9 via the RAS/MEK/ERK and the PI3K/AKT signaling^4^,^5^. Our unpublished data showed that DDX3 may activate PI3K/AKT signaling to promote β-catenin activation by increasing its protein stability due to phosphorylation of GSK3β. Therefore, activation of the CK1ε/Dvl2 axis by DDX3 may play a partial role in β-catenin/TCF activation during colorectal tumorigenesis.

In summary, we have provided evidence that DDX3 promotes cell invasiveness and xenograft lung tumor nodules formation via the CK1ε/Dvl2 axis due to activation of β-catenin/TCF signaling. The formation of xenograft lung tumor nodules was significantly suppressed by an inhibitor of CK1ε (PF4800567) or β-catenin/TCF signaling (XAV939). The expression of pDvl2 and nuclear β-catenin mediated by DDX3 was associated with poor outcomes in colorectal cancer patients. Therefore, we suggest that CK1ε or the β-catenin/TCF signaling might be potential targets for improvement of tumor regression and outcomes, particularly in patients who harbor high-DDX3/high-nuclear β-catenin or high-DDX3/high-pDvl2/high-nuclear β-catenin tumors.

## Materials and Methods

### Study subjects

This study consisted of 75 patients with colorectal cancer. All patients were unrelated ethnic Chinese and residents of Central Taiwan. The inclusion criteria for patients were: primary diagnosis with colorectal carcinoma; no metastatic disease at diagnosis; no previous diagnosis of carcinoma; no neoadjuvant treatment before primary surgery; no evidence of disease within one month of primary surgery. Tumor specimens collected from surgically resected colorectal cancer patients were stored at 80°C at the Division of Colon and Rectum, Chung Shan Medical University Hospital (Taichung, Taiwan, ROC), between 1994 and 2006. The present experiments were conducted in accordance with the Declaration of Helsinki^25^. The protocol in this study was approved by the Ethics Committee at the Chung Shan Medical University Hospital (CS07159), and written informed consent was obtained from the patients prior to study enrollment. The tumor type and stage of each collected specimen were histologically determined according to the WHO classification system. Cancer relapse data were obtained by chart review and confirmed by surgeons. Clinical parameters and information were collected from chart review and the Taiwan Cancer Registry, Department of Health, Executive Yuan, Taiwan, ROC. Survival time was defined as the period from the date of primary surgery to the date of death. The median follow-up time was 1165 days (ranging from 23 to 2479 days) and the end of the follow-up period was December 2007. Based on the follow-up data, relapse data from 75 patients were available, indicating that 20 patients relapsed (16 had distant metastasis, and 4 had local and distant metastasis). Tumors frequently relapsed in the liver (9 patients) and metastasized in the lung (2 patients), hypopharynx (1 case), bone (1 case), left paraaortic lymph node (1 case), pelvis (1 case), and rectum (1 case). Four patients had tumors that metastasized to more than one organ.

### Cell lines

CCM2 cells were primary cultures from Taiwanese colorectal cancer patients and were kindly provided by Dr. Wun-Shaing Wayne Chang (National Institute of Cancer Research, National Health Research Institutes, Miaoli, Taiwan). The T84, HCT116, and HCT15 cells were obtained from the American Type Culture Collection (ATCC) and were cultured under conditions described elsewhere and stored according to the suppliers’ instructions. The cells were used at passages 5 to 20. Once resuscitated, cell lines were routinely authenticated (once every 6 months, cells were last tested in December 2013) through cell morphology monitoring, growth curve analysis, species verification by isoenzymology and karyotyping, identity verification using short tandem repeat profiling analysis, and contamination checks. The CCM2, HCT15, and T84 cell lines harbored KRAS, APC, and p53 mutations, but possessed a wild-type β-catenin gene. The HCT116 cells harbored KRAS and β-catenin mutations, but possessed wild-type APC and p53 genes. [CCM2: KRAS (G12V); APC (Q1338X(STOP codon)); p53 (R273H). HCT15: KRAS (G13D); APC (Q2166X(STOP codon), I1417fsX2); p53 (S241F; C1101-2A>C); T84: KRAS (G13D); APC (L1488fs*19); p53 (P60L; C376-1G>T). HCT116: KRAS (G13D); β-catenin (S45del)].

### Chemicals and antibodies

XAV939 was obtained from Selleckchem (Houston, TX, USA). PF4800567 was purchased from Sigma Chemical (St. Louis, MO, USA). Anti-PP2A, Anti-CK1εand anti-Dvl2 antibodies were obtained from GeneTex (Irvine, CA, USA). Phosphorylated Dvl2 (S143) antibody were purchased from Abcam (Cambridge, MA, USA). Phosphorylated β-catenin (S33, S37 and T41), GSK-3β, and phosphorylated GSK-3β (S9) antibodies were purchased from Cell Signaling (Danvers, MA, USA). All other antibodies were purchased from Santa Cruz Biotechnology (Dallas, TX, USA).

### Plasmid constructs and transfection

DDX3 (#1 TRCN0000000001; #2 TRCN0000000003), β-catenin (TRCN0000314991), CK1ε (TRCN0000009965), and Dvl2 (TRCN0000033340) shRNAs were purchased from the National shRNA Core Facility, Academia Sinica, Taiwan, ROC. DDX3 overexpression plasmids were provided from Addgene (Addgene Company, Cambridge, MA, USA). The TOP flash plasmid (a reporter plasmid containing multiple copies of wild-type TCF-binding sites) was purchased from Millipore (Billerica, MA, USA). Different concentrations of expression plasmids were transiently transfected into lung cancer cells (1 **×** 10^6^) using the Turbofect reagent (Glen Burnie, MD, USA). After 48 hours (h), the cells were harvested and whole cell extracts were assayed in subsequent experiments.

### Luciferase reporter assay

Cells were transfected with the indicated combination of reporter plasmids with overexpression and knockdown plasmids. Luciferase assays were performed using the Luciferase Reporter Assay System (Promega, Madison, WI, USA) 24 h after transfection. Normalized luciferase activity was reported as the ratio of luciferase activity/β-galactosidase activity.

### Western blotting

For immunoblotting, cell lysates were prepared as described previously^26^. Equal amounts of protein were separated onto sodium dodecyl sulfate-polyacrylamide gel electrophoresis (SDS-PAGE) gels and then transferred from the gel onto a polyvinylidene difluoride membrane (PerkinElmer, Norwalk, CT, USA). After blocking, the membranes were reacted with antibody at 4 °C overnight, followed by incubation with horseradish peroxidase-conjugated secondary antibody for 1 hour. The blots were observed using an enhanced chemiluminescence kit (PerkinElmer).

### Immunohistochemistry analysis

The immunohistochemical procedures and quantification methods were as described previously^27^. The intensities of the signals were evaluated independently by three observers. Immunostaining scores were defined as the cell staining intensity (0 = nil; 1 = weak; 2 =moderate; and 3 = strong) multiplied by the percentage of labeled cells (0–100%), leading to scores from 0 to 300. A score over 150 was rated as “high” immunostaining, while a score less than 150 was rated as “low.” The cytoplasmic expression and nuclear expression of β-catenin were separately examined by at least two observers^28^. High-nuclear β-catenin tumors were defined as high-nuclear β-catenin/high-cytoplasmic β-catenin (N+/C+) and high-nuclear β-catenin/low-cytoplasmic β-catenin N+/C−; otherwise, low-nuclear β-catenin tumors were defined as N−/C− and N−/C+.

### Invasion assay

A Boyden chamber with a pore size of 8 μm was used for the *in vitro* cell invasion assay. Cells (1 × 10^4^) in culture medium (HyClone, Ogden, UT, USA) containing 0.5% serum were plated in the upper chamber and 10% fetal bovine serum was added to culture medium in the lower chamber as a chemoattractant. The upper side of the filter was covered with 0.2% Matrigel (Collaborative Research, Boston, MA, USA) diluted in RPMI-1640. After 16 h, cells on the upper side of the filter were removed and cells that adhered to the underside of membrane were fixed in 95% ethanol and stained with 10% Giemsa dye. The number of invasive cells was counted by examining 10 contiguous fields of each sample to obtain a representative number of cells that had invaded across the membrane.

### 3-(4,5-cimethylthiazol-2-yl)-2,5-diphenyl tetrazolium bromide (MTT) assay

The cell lines were cultured in 96-well flat-bottomed microtiter plates supplemented with RPMI 1640 or DMEM (containing 10% heat-inactivated fetal bovine serum, 100 units/mL penicillin, and 100 units/mL streptomycin) at 37 °C in a humidified incubator in an atmosphere of 95% air and 5% CO_2_. After the indicated incubation, the *in vitro* effects of these treatments were determined by the MTT assay (at 570 nm).

### *In vivo* tail-vein injection animal model

The tail*-*vein injection animal model was conducted as previously reported^29^,^30^,^31^. The procedures for therapeutic experiments were according to previous reports with slight modification^32^,^33^. Mice were injected intraperitoneally with vehicle control (saline), XAV939 (5 mg/kg), or PF4800567 (5 mg/kg) (n = 5 per group) daily from day 1 to day 14. Mice were injected with T84 VC and DDX3-overexpressing stable clone via the tail vein (10^5^ cells in 0.1 mL of PBS) at day 7. Six weeks after injections, the mice were euthanized and the lungs were dissected and examined for the development of visible lung tumor nodules. Tissues were processed for hematoxylin and eosin staining. Animal care and experimental procedures were performed with approval from the Institutional Animal Care and Use Committee of Taipei Medical University (LAC-2013-0205) in accordance with the National Institutes of Health Guide for the Care and Use of Laboratory Animals (NIH publication number 8023, revised 1978).

### Statistical analysis

Statistical analysis was performed using the SPSS statistical software program (Version 18.0; SPSS Inc., USA). The association between DDX3, pDvl2, and β-catenin protein expression was analyzed by the chi-square test. Survival plots were generated using the Kaplan-Meier method and differences between patient groups were determined by the log-rank test. Multivariate Cox regression analysis was performed to determine OS and RFS. The analysis was stratified for all known variables (age, gender, smoking status, and tumor stage) and protein expression.

## Additional Information

**How to cite this article**: He, T.-Y. *et al.* DDX3 promotes tumor invasion in colorectal cancer via the CK1ε/Dvl2 axis. *Sci. Rep.* **6**, 21483; doi: 10.1038/srep21483 (2016).

## Supplementary Material

Supplementary Information

## Figures and Tables

**Table 1:** Correlation of DDX3 with pDvl2 and nuclear β-catenin expression and the association between pDvl2 and nuclear β-catenin in tumors from colorectal cancer patients.

Variables	Case No.	pDvl2			Nuclear β-catenin*		
		Low (%)	High (%)	P	Low (%)	High (%)	P
DDX3							
Low	44	30 (68)	14 (32)	0.001	29 (66)	15 (34)	0.004
High	31	9 (29)	22 (71)		10 (32)	21 (68)	
pDvl2							
Low	39				36 (92)	3 (8)	< 0.001
High	36				3 (8)	33 (92)	

The number in the parentheses was the percentage of patients in the category (%).

^*^High-nuclear β-catenin tumors were defined as high-nuclear β-catenin/high-cytoplasmic β-catenin (N+/C+) and high-nuclear β-catenin/low-cytoplasmic β-catenin N+/C−; otherwise, low-nuclear β-catenin tumors were defined as N−/C− and N−/C+.

**Table 2:** Cox regression analysis for the prognostic value of DDX3, pDvl2, nuclear β-catenin, and their combinations on OS and RFS in colorectal cancer patients.

Variables	OS				RFS			
	Case No.	Adjusted HR	95% CI	P	Case No.	Adjusted HR	95% CI	P
DDX3								
Low	44	1			44	1		
High	31	2.57	1.19–5.56	0.016	31	2.26	1.14–4.45	0.019
pDvl2								
Low	39	1			39	1		
High	36	3.07	1.34–7.05	0.008	36	2.79	1.35–5.75	0.005
Nuclear β-catenin*								
Low	39	1			39	1		
High	36	3.97	1.71–9.26	0.001	36	2.96	1.43–6.12	0.004
**DDX3/pDvl2**								
Others	53	1			53	1		
High/high	22	4.55	2.01–10.29	<0.001	22	2.71	1.37–5.36	0.004
**DDX3/Nuclear β-catenin**								
Others	54	1			54	1		
High/High	21	5.61	2.49–12.65	<0.001	21	2.67	1.35–5.27	0.005
**DDX3/pDvl2/Nuclear β-catenin**								
Others	55	1			55	1		
+/+/+	20	4.78	2.13–10.72	<0.001	20	2.41	1.22–4.78	0.012

OS: overall survival; RFS: relapse free survival; HR: haozard ratio.

^*^High-nuclear β-catenin tumors were defined as high-nuclear β-catenin/high-cytoplasmic β-catenin (N+/C+) and high-nuclear β-catenin/low-cytoplasmic β-catenin N+/C−; otherwise, low-nuclear β-catenin tumors were defined as N−/C− and N−/C+.
