# Supplementary material for: DDX3 promotes tumor invasion in colorectal cancer via the CK1ε/Dvl2 axis
Source: Sci Rep. 2016 Feb 19;6:21483. doi: 10.1038/srep21483 (PMC4759588; doi:10.1038/srep21483)
Supplement: Supplementary Information [file srep21483-s1.doc]

**DDX3 promotes tumor invasion in colorectal cancer via the CK1ε/Dvl2 axis**

Tsung-Ying He^1^, De-Wei Wu^2^, Po-Lin Lin^1^, Lee Wang^3^, Chi-Chou Huang^4,5^, Ming-Chih Chou^1,4,5^ and Huei Lee^2^

**Supplementary Figure S1.** Full-length blots for main figures corresponded to Fig. 1a, 1c, 2a and 2b.

**Supplementary Figure S2.** Full-length blots for main figures corresponded to Fig. 2c and 3a.

**Supplementary Figure S3.**  **Effects of PF4800567 and XAV939 on the cell proliferation of DDX3-overexpressing T84 cells** T84 cells were transfected with DDX3-overexpression plasmid. After 24 h, these cells were treated with a CKlε inhibitor (PF4800567) or a β-catenin inhibitor (XAV939) for an additional 5 h, and then the cell viability for indicated cells was determined by MTT assays at 0 h and 16 h. The relative cell viability of the VC cells was arbitrarily assigned as 1 and the standard deviation was shown by error bars.

**Supplementary Figure S1.**

**
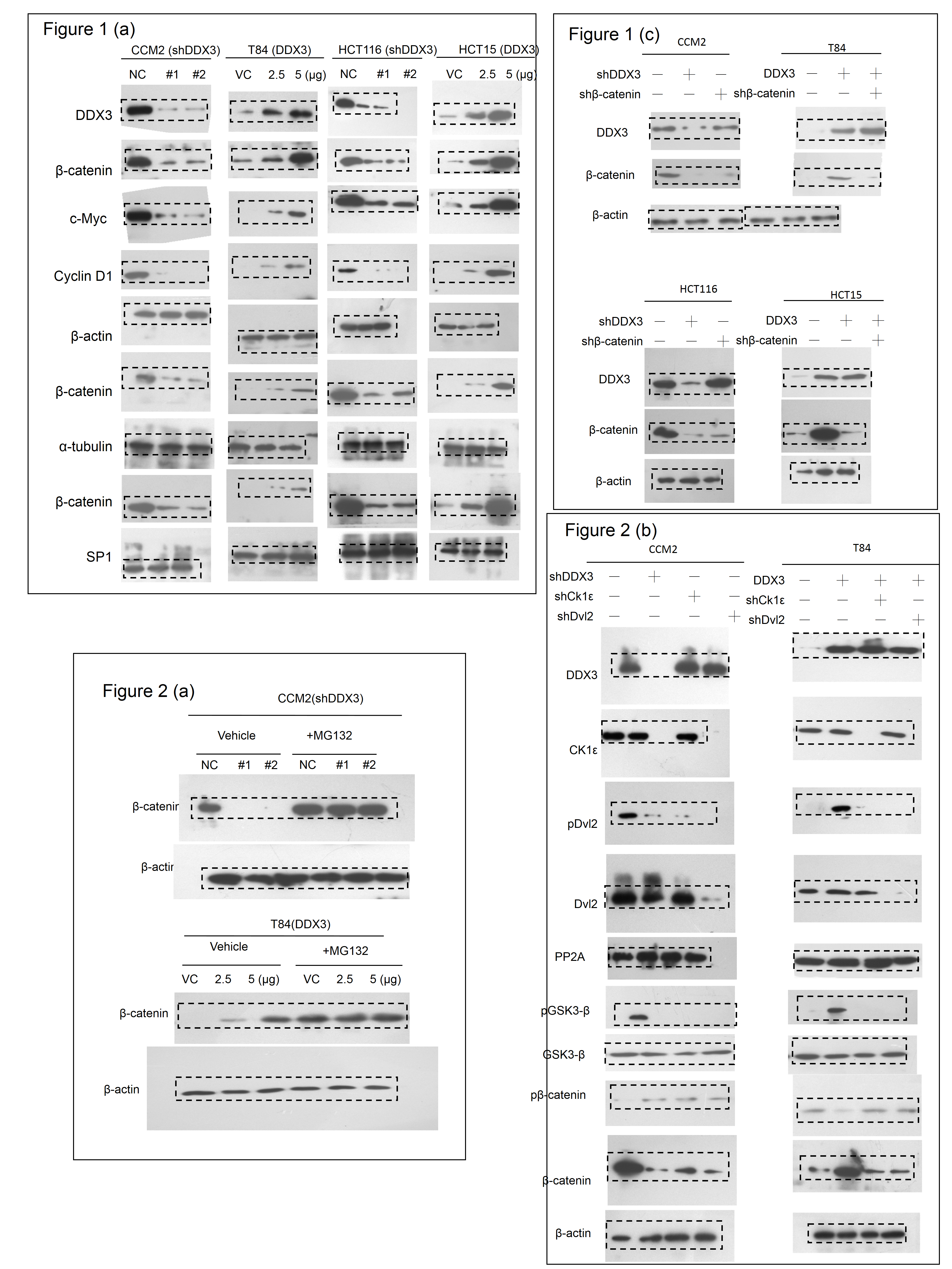
**

**Supplementary Figure S2.**

**
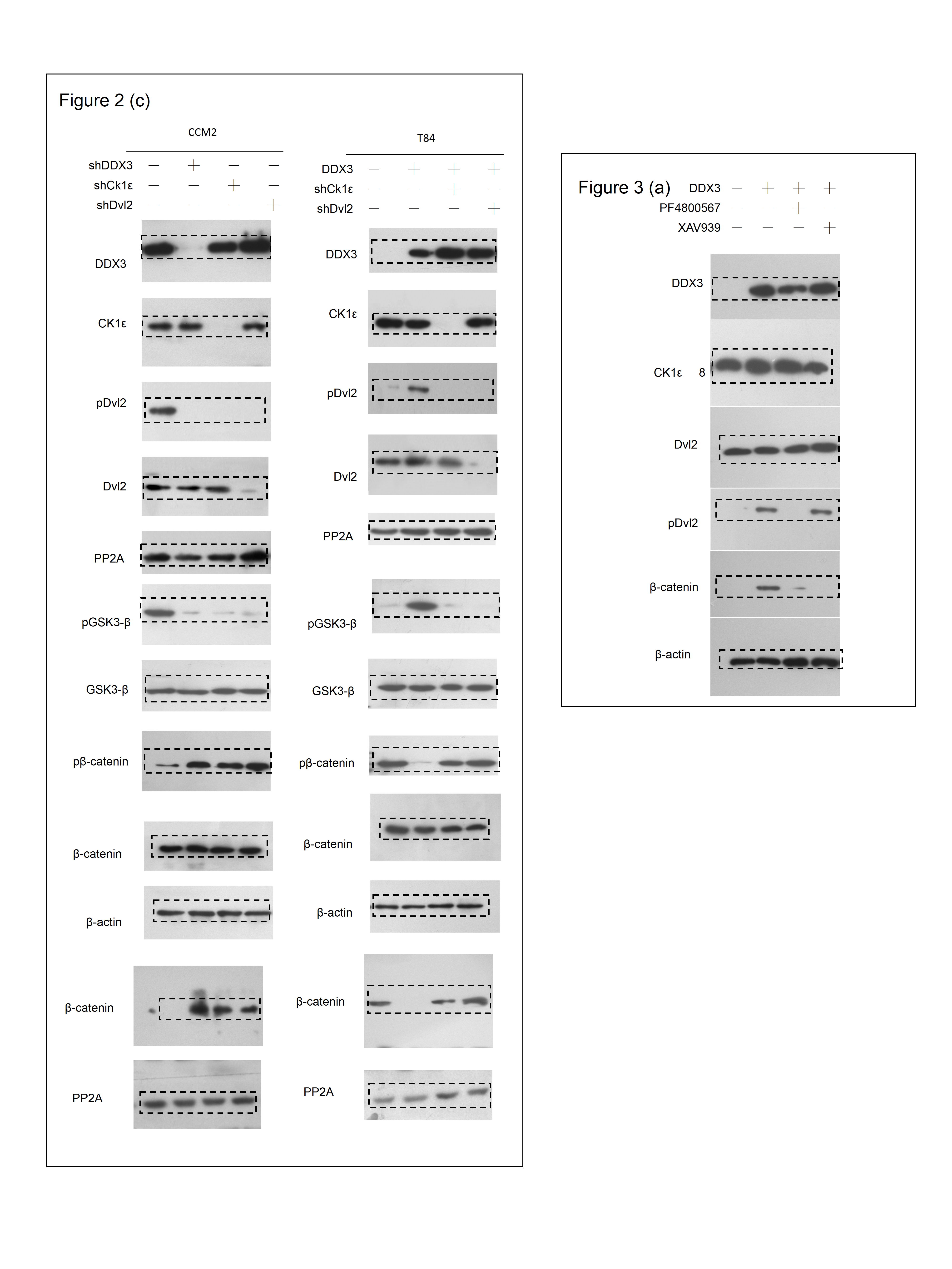
**

**Supplementary Figure S3**
